# Enhancing Part-to-Part Repeatability of Force-Sensing Resistors Using a Lean Six Sigma Approach

**DOI:** 10.3390/mi13060840

**Published:** 2022-05-27

**Authors:** Andrés O. Garzón-Posada, Leonel Paredes-Madrid, Angela Peña, Victor M. Fontalvo, Carlos Palacio

**Affiliations:** 1 Faculty of Engineering, Universidad Católica de Colombia, Carrera 13 # 47-30, Bogota 110221, Colombia; aogarzon@ucatolica.edu.co (A.O.G.-P.); vmfontalvo@ucatolica.edu.co (V.M.F.); 2 Department of Applied Physics, Materials and Surface Lab (Nanotechnology Unit), Faculty of Sciences, Universidad de Málaga, ES29071 Malaga, Spain; 3 Faculty of Mechanical, Biomedical and Electronic Engineering, Universidad Antonio Nariño, Carrera 7 # 21-84, Tunja 150001, Colombia; angelapena@uan.edu.co; 4 GIFAM Group, Faculty of Sciences, Universidad Antonio Nariño, Carrera 7 # 21-84, Tunja 150001, Colombia; carlospalacio@uan.edu.co

**Keywords:** nanocomposites, tactile sensors, force sensors, pressure sensors, sensor phenomena and characterization, regression analysis, Gaussian distribution

## Abstract

Polymer nanocomposites have found wide acceptance in research applications as pressure sensors under the designation of force-sensing resistors (FSRs). However, given the random dispersion of conductive nanoparticles in the polymer matrix, the sensitivity of FSRs notably differs from one specimen to another; this condition has precluded the use of FSRs in industrial applications that require large part-to-part repeatability. Six Sigma methodology provides a standard framework to reduce the process variability regarding a critical variable. The Six Sigma core is the DMAIC cycle (Define, Measure, Analyze, Improve, and Control). In this study, we have deployed the DMAIC cycle to reduce the process variability of sensor sensitivity, where sensitivity was defined by the rate of change in the output voltage in response to the applied force. It was found that sensor sensitivity could be trimmed by changing their input (driving) voltage. The whole process comprised: characterization of FSR sensitivity, followed by physical modeling that let us identify the underlying physics of FSR variability, and ultimately, a mechanism to reduce it; this process let us enhance the sensors’ part-to-part repeatability from an industrial standpoint. Two mechanisms were explored to reduce the variability in FSR sensitivity. (i) It was found that the output voltage at null force can be used to discard noncompliant sensors that exhibit either too high or too low sensitivity; this observation is a novel contribution from this research. (ii) An alternative method was also proposed and validated that let us trim the sensitivity of FSRs by means of changing the input voltage. This study was carried out from 64 specimens of Interlink FSR402 sensors.

## 1. Introduction

Force-sensing resistors (FSRs) are typically manufactured from a blend of an insulating polymer with conductive nanoparticles ([1,2,3,4,5,6]. The resulting nanocomposite exhibits a piezoresistive response that can be used to either measure compressive forces [6] or tensile loads [3,4,7]. Given the low profile and low cost of FSRs, their usage in research and industrial applications is currently increasing [8]. Multiple studies related to gait analysis [9,10,11], robotics [12,13], and other disciplines have reported the usage of polymer nanocomposites to perform strain/stress measurements [14,15,16,17]. 

In recent years, there has been an explosion in the number of studies that have developed novel techniques for manufacturing conductive polymers composites (CPCs). Recent studies have incorporated conductive polymers as a replacement for conductive particles [18,19,20]; this is a desirable characteristic given the toxicity of graphene, carbon nanotubes (CNTs), and carbon black (CB) [21]. Similarly, the introduction of self-healing properties to the nanocomposite has been also studied by multiple authors [22,23,24]. Previous authors’ works have mainly focused on: first, improving the performance of FSRs by studying the effect of source voltage in the repeatability of measurements [25], and second, developing tailored driving circuits that can help us to minimize time drift and electrical hysteresis [26].

Despite the previously mentioned efforts, the overall performance of FSRs and CPCs still lags behind that of load cells performance in multiple ways. By comparing commercial FSRs [27,28,29] with an LCHD-5 load cell from Omega Engineering (Norwalk, CT, USA) [30], a difference of up to two orders of magnitude is evident in the metrics of hysteresis and accuracy. Fortunately, studies from Urban et al. [31] and Nguyen and Chauban [32] have helped to narrow the performance gap between both sensing technologies. However, one of the most important drawbacks of FSRs, which has not been yet addressed by specific literature, is the inability to know sensor sensitivity a priori. Given the random dispersion of conductive nanoparticles along the insulating polymer matrix [33], it is not possible to determine the resulting sensitivity of a given nanocomposite, i.e., every specimen has a different sensitivity. This characteristic limits the extensive usage of FSRs since individual sensor calibration is required before use. This condition is not a major concern when only a few sensors are required in the final application, but when multiple sensors are required, sensor characterization is a time-consuming task; ultimately, a more suitable sensing technology may be preferred instead. Robotic skins and tactile pads are representative examples of applications requiring multiple sensing points. In these types of deployments, sensor arrays with multiple tactels are employed to detect shapes and force profiles [34].

As pointed out by Castellanos-Ramos et al., the characterization of piezoresistive tactile pads required a complex test bench with an air compressor and tailored hardware; this was performed to match the specific dimensions of the tactile pad [34]. The aim of this study is to develop techniques that save time and resources by avoiding individual sensor calibration. In this research, we address such a concern by using the Lean Six Sigma Methodology (LSSM) to a group of 64 specimens of commercial FSRs, manufactured by Interlink Electronics, Inc. (Westlake Village, CA, USA) [27].

It must be stated that the application of the LSSM to FSRs represents a novelty. By looking up the following keyword combinations in the Scopus search engine without year constraints: Six Sigma and FSR, Six Sigma and polymer composite, Six Sigma and piezoresistive sensor, a total of 68 entries were found. Most of the entries found were inaccurate because Sigma is used to designate sensors’ sensitivity. Only six studies truly reported the use of LSSM [35,36,37,38,39,40], but most of them reported calibration procedures for pressure-sensing equipment in automotive applications.

The rest of this paper is organized as follows: Section 2 briefly describes the theoretical foundations of the Lean Six Sigma methodology and the physical modeling of FSRs, the experimental setup for gathering sensor data is described in Section 3, followed by the application of the LSSM in Section 4. Conclusions are addressed in Section 5.

## 2. Theoretical Foundations

### 2.1. Application of the Six Sigma Methodology

Readers may refer to Appendix A for a theoretical description of the Lean Six Sigma methodology (LSSM). In this section, we describe the application of the LSSM to our study case. The goal of this research is to reduce the sensitivity dispersion of FSRs by adjusting their driving voltage and/or by discarding noncompliant sensors. As previously mentioned, the reduction in sensitivity dispersion avoids individual sensor calibration, which ultimately saves time and resources.

In order to reduce sensitivity dispersion, we have deployed the core of the LSSM, i.e., the DMAIC cycle (Define, Measure, Analyze, Improve, and Control). The Six Sigma methodology can be implemented during any phase of product development, manufacturing, and later deployment into the final application [41]. The current research only considered the application of the LSSM to reduce FSR variability, as measured from their sensitivity; this is known in industry as the enhancing of part-to-part repeatability [27]. In this study, we only considered compensation techniques into the last stage of final application deployment. However, several authors have explored different methods during sensor fabrication, such as addition of surfactants [42] and the application of magnetic fields to reduce the percolation threshold [43]. Given the fact that we only applied compensation techniques into the last stage of final application deployment, the ultimate goal of Six Sigma reduction was partially achieved.

In order to obtain a Six Sigma reduction in sensitivity dispersion, it is required to apply the DMAIC cycle from the reception of raw materials, followed by rigorous control of sensor manufacturing and assembly; this whole process, although possible, would require separate research.

In this study, the DMAIC was deployed by measuring the output voltage at null force (*U_o_^0N^*) for each sensor as received from the manufacturer. Later, we correlated *U_o_^0N^* with sensor sensitivity and developed a statistical model to trim the driving voltage of the FSRs; this process required a thorough understanding of the sensing mechanism of FSRs, which are discussed in Section 2.2. Specific details of the DMAIC cycle are later addressed in Section 4.

### 2.2. Physical Modeling of Force-Sensing Resistors

Multiple authors have studied the underlying physics of CPCs under different mechanical and electrical conditions, such as compressed/uncompressed operation [44,45,46], sourcing at low/high voltages [47,48], and finite element analysis by considering changes in particle dimension and spatial distribution [49,50]. The aforementioned studies agree on the fact that piezoresistivity mainly originates from two phenomena: quantum tunneling occurring among adjacent conductive particles separated by the insulating polymer, and constriction resistance occurring at clusters of multiple particles. Each phenomenon is subsequently described.

#### 2.2.1. Quantum Tunneling as a Source of Piezoresistivity

This conduction mechanism can be explained from widely known equations by Simmons that describe the tunneling current between electrodes separated by a thin insulating film [51]. When operating at voltages near zero, a tunneling barrier of thickness (*s*), with an input applied voltage (*U*), exhibits a current density (*J*) equal to:

(1) $$J(U,s)=\frac{3e^{2}\sqrt{2mV_{a}}}{2h^{2}s}U\exp\left(-\frac{4\pi s}{h}\sqrt{2mV_{a}}\right)$$

where (*h*) is the Planck constant, (*V_a_*) is the height of the insulating potential barrier and (*m*), (*e*) are the electron mass and charge, respectively. However, if *U >> V_a_/e*, the current density can be obtained from the following expression:

(2) $$J(U,s)=\frac{2.2e^{3}U^{2}}{8\pi hV_{a}s^{2}}\exp\left(-\frac{8\pi s}{2.96heU}\sqrt{2mV_{a}{}^{3}}\right)$$

For the sake of this paper, the full set of equations for the intermediate voltage ranges are not presented since they are not required, but they can be found in the original study from Simmons [51]. Note that regardless of the applied voltage, *U*, current density changes in a negative exponential fashion with the interparticle separation, i.e., this observation also holds for the intermediate voltage equations not included in this study. Similarly, when operating at high input voltages, a change in *U* impacts current density (and also the sensor’s sensitivity) in a nonlinear fashion. Under a compression regime, it is possible to relate *s* with the external applied force (*F*) as next:

(3) $$s(F)=s_{o}(1-F/(AM))$$

where (*s_o_*) is the uncompressed interparticle separation, (*A*) is the sensor area, and (*M*) is the compressive modulus of the nanocomposite. It is possible to substitute (3) into (1) and (2) to obtain unified equations that relate current density with the external applied force.

**Figure 1:**
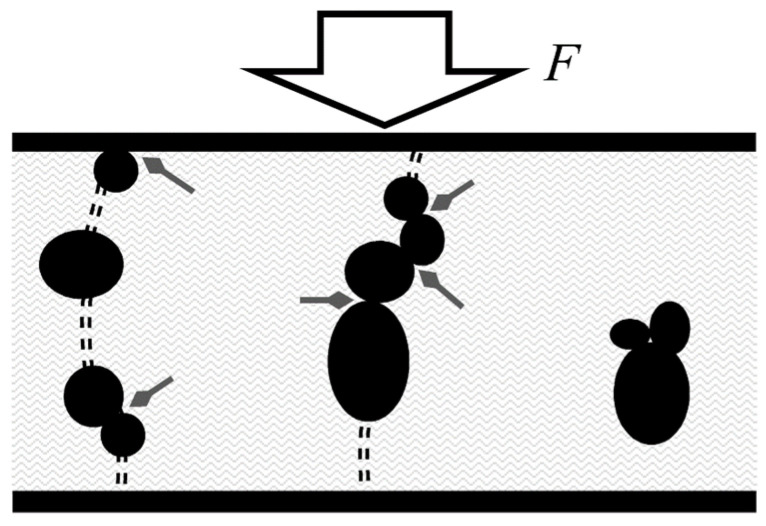
Sketch of a nanocomposite comprising randomly spaced conductive particles in an insulating polymer matrix; the material is sandwiched between metallic electrodes and subjected to an external compressive force (*F*). Quantum tunneling conduction is shown as double dashed line paths; they connect isolated particles, thus creating the tunneling resistance. Greyed diamond arrows mark the constriction resistance occurring between adjacent particles (as well as between electrode and particles in contact); they create the constriction resistance (*R_c_*). Particles located too far from each other fail to create a conduction path.

Figure 1 shows the tunneling phenomenon occurring in multiple spots along the nanocomposite. In practice, the net tunneling resistance (*R_tun_*) is originated from the multiple parallel and series connections that occur in the 3D polymer matrix. Unfortunately, an explicit expression for *R_tun_* can only be found for voltages near zero using (1), but when *U* >> *V_a_*/*e*, an explicit relationship for *U*/*J* cannot be found since *U* appears as part of the argument in the exponential function, see (2).

For most CPCs involving conductive nanoparticles, the height of the potential barrier is, at most, 0.57 eV for a Sn–Pb/PS nanocomposite [52], where PS stands for polystyrene. Later, in Section 4, we demonstrate that the optimal operating range for the Interlink sensors is accessible for voltages around 3 V. By taking the largest case of *V_a_* = 0.57 eV, we can straightforwardly discard (1) as a valid model since it only holds for voltages near 0 V. On the other hand, Equations (2) and (3) are better suited to modeling the piezoresistive response of Interlink sensors when predominantly operating under quantum tunneling regime; this occurs because (2) holds when *U* >> *Va*/*e*, which is the working case since *U* is around 3 V and *V_a_* = 0.57 eV.

Finally, it must be clarified that current density can be converted to current by considering the effective tunneling area. Nonetheless, the effective tunneling area is not the same as the sensor area, *A*, because electrons flow only through some regions of the polymer with high particle concentration; see Figure 1. A comprehensive discussion of Simmons’s equations for modeling piezoresistive sensors was performed by the authors in a previous study [25]. Such study experimentally determined the effective tunneling area, as well as the whole set of parameters considered in (1)–(3).

#### 2.2.2. Constriction Resistance as a Source of Piezoresistivity

The constriction resistance (*R_c_*) originates at two different spots: first, at the incomplete percolation paths located along the polymer matrix; these are the particle-particle interactions, and second, at the sensor boundary where electrode-particle interactions occur; both cases are shown in Figure 1. For particles with diameters ranging from a hundred nanometers to tens of micrometers, the contact size is comparable with the mean free path of electrons, thus causing a restriction to free electron motion [53]. According to the model developed by Mikrajuddin et al. [54], when operating under compression regime, the constriction resistance changes in an inversely proportional fashion with the applied force:

(4) $$R_{c}(F)\propto R_{o}/F$$

The constant (*R_o_*) depends on the particles physical dimensions, Poisson ratio of the material, and elasticity modulus. For simplicity purposes, the exact expression is not presented here as it comprises a piecewise function for the elastic and inelastic interaction occurring at the interface; such an explanation falls out the scope of this article. However, we must emphasize that the constriction resistance is a *voltage-independent* phenomenon.

By recalling Figure 1, we note that *R_c_* originates at multiple spots along the nanocomposite, therefore, we can only measure the net contribution of the multiple series-connected and parallel-connected constriction resistances, i.e., *R_c_* forms an intricate network of resistances. The net contribution of the multiple constriction resistances, *R_c_*, is henceforth designated as the contact resistance (*R_con_*). We will no longer use *R_c_* in this manuscript.

#### 2.2.3. Combining Tunneling and Contact Resistances

Sensor resistance (*R_FSR_*) is calculated by summing the contribution of the tunneling and the contact resistance as follows:

*R_FSR_*
 = 
*R_tun_*
 + 
*R_con_* (5)

Equation (5) was initially proposed by Kalantari et al. [45], and was later embraced by the authors [25].

As demonstrated in the next section, the random dispersion of conductive nanoparticles creates a specific sensor response in which either *R_tun_* or *R_con_* dominates for a given specimen. If particles are grouped in clusters separated by the insulating polymer, then *R_tun_* dominates, but if particles are in direct contact (forming percolation paths), then *R_con_* dominates. A discussion regarding the influence of *U* over *R_tun_* and *R_con_* can be found in previous works [25].

## 3. Materials and Methods

In previous work [55], the experimental setup was thoroughly described; therefore, only a brief description is presented here.

### 3.1. Mechanical Setup

The mechanical setup comprised a tailored test bench capable of handling up to 16 Interlink FSR402 sensors simultaneously; this let us speed up the characterization process by avoiding single-sensor measurement [55]. Forces were applied from a linear motor and a spring. An LCHD-5 load cell was deployed to close the force loop [30]. 

### 3.2. Electrical Setup

**Figure 2:**
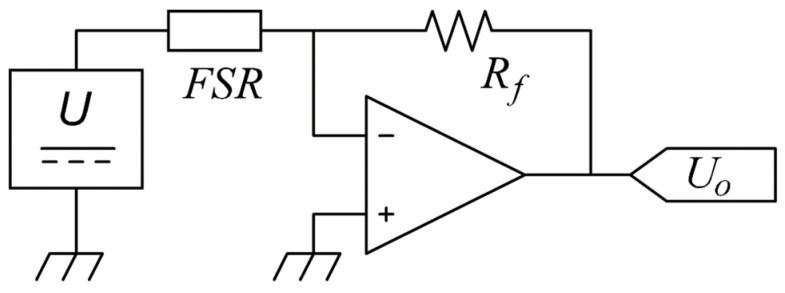
Electrical setup for driving the FSRs. The input voltage (*U*) was implemented from a digital-to-analog converter (DAC) to enable sensor characterization at multiple voltages.

An amplifier in inverting configuration was used as the interface circuit; see Figure 2. This setup was preferably chosen over a voltage divider because the amplifier let us control the voltage across the sensor at any time. Analog multiplexers were deployed to enable time-multiplexed readings of the 16 FSRs. From the amplifier output voltage (*U_o_*), we could determine sensor resistance, *R_FSR_*, given the amplifier model:

(6) $$U_{o}=-(R_{f}/R_{FSR})\cdot U$$

From (6), we can either use *R_FSR_* or *U_o_* as the variable to measure. However, *U_o_* was preferably chosen over *R_FSR_*, because the former exhibited a linear relationship with the applied force, *F*. The feedback resistor (*R_f_*) was set to 560 Ω for all the experimental tests in this study. The acquisition system comprised a 16-bit analog-to-digital converter (ADC) model NI-9205 and a 16-bit digital-to-analog converter (DAC) model NI-9263; the former was required for measuring *U_o_*, whereas the latter was employed to generate multiple *U* values. The system controller was the CRIO-9035 running LabVIEW Real Time.

### 3.3. Methods 

Each step of the DMAIC cycle is briefly described in Table 1. A thorough description of the process is presented in the rest of the manuscript.

Given the DMAIC cycle, we *defined* sensor sensitivity (*m*) as the variable to study with units of volts per Newton. The sensitivity *measurements* were performed at multiple input voltages as described in Table 1. From the 64 sensors at 19 different voltages, a total of 1216 sensitivities were collected. No lot control was considered for the 64 sensors; this is important to note because the manufacturer has stated different part-to-part repeatability for single-lot sensors [27]. Given the setup of Figure 2, sensitivity was obtained from a least-squares fitting process with general formula:

(7) $$U_{o}=mF+b$$

where (*b*) was the y-intercept resulting from the fit. It must be clarified that *b* was not considered in this study; instead, we measured the output voltage at 0 N (*U_o_^0N^*). The reasoning behind this decision is provided in the next step. The *analysis* stage comprised an extensive evaluation of the experimental data in perspective of the theoretical foundations from Section 2. Before discussing the experimental data, we had to develop the following theoretical *claims*:

(i) Regardless of *U*, larger compressive forces increase the current density as the interparticle separation is reduced and the contact resistance is lowered; see (1)–(5). (ii) For null applied force, incremental *U* yields larger current density, thus *U_o_^0N^* is increased as well; see (2). However, we must recall that the constriction resistance is a voltage-independent phenomenon, thus changing *U* does not modify *R_con_*; see (4). (iii) By taking the derivative of *J(U,s(F)*) with respect to *F*, we note that for the same force profile, the derivative increases for larger *U*. The rate of change between *J(U,s(F))* and *F* determines sensor sensitivity as it relates the change in sensor current with the input force; this statement only applies for the tunneling resistance. Claim (iii) can be summarized as follows: larger *U* yields greater sensitivity, whereas reducing *U* diminishes sensor sensitivity.

(iv) It is clear from Figure 1 that both piezoresistive phenomena occur simultaneously under any applied stress. Nonetheless, the experimental data support the hypothesis that one phenomenon usually dominates over the other for a given sensor. For example: when the contact resistance dominates, the percolation paths are the main source of piezoresistivity; whereas quantum tunneling is less important. Under this scenario, *U_o_^0N^* is large but m is small. To understand this, we must compare (2) and (3) with (4) as follows: given the predominant percolation paths along the FSR, a large *U_o_^0N^* is naturally expected because these paths tend to easily transport electrons from one electrode to the other, however, when an external force is applied, the change in resistance is small as predicted by (4), i.e., the contact resistance changes with the inverse of the applied force. In contrast, when quantum tunneling dominates, the percolation paths are incomplete or nonexistent; in this case, (2) and (3) instead play a major role. Under this scenario, a small increment in *s*—caused by an external force—results in a dramatic change in current density due to the exponential dependence in (2) and (3); this can be understood as a large sensitivity. Nonetheless, when quantum tunneling dominates and the sensor is unloaded, *U_o_^0N^* is small because there are not percolation paths that connect both electrodes.

Experimental results supporting previous statements are next presented. Thereafter, *improve* and *control* stages are described.

## 4. Results 

**Figure 3:**
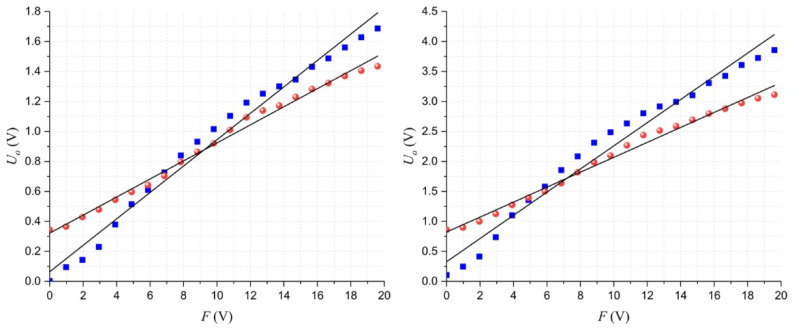
Plots of the output voltage (*U_o_*) as a function of the applied force (*F*) for two different specimens at different input voltages (*U*). The fit to (7) has been overlaid in both cases. The sensor where percolation dominates is shown with red spherical markers; the sensor where quantum tunneling dominates is shown with blue square markers. Data taken at (**left**) *U* = 3 V and (**right**) *U* = 6 V.

Figure 3 shows the experimental data for two specimens at different input voltages. The claims (i)–(iv) from Section 3 can be validated with the results reported in this figure, as follows: (i) larger forces increase *U_o_*; (ii) for a given sensor, increasing *U*, also increases *U_o_^0N^*, and similarly, (iii) larger *U* increases *m* as well. Finally, (iv) if *U_o_^0N^* is large, then percolation dominates and *m* is usually small. The opposite case can be also observed in Figure 3; if *U_o_^0N^* is small, then quantum tunneling dominates, and *m* is generally large.

From the experimental data resulting at each input voltage, we obtained sensor sensitivity for each specimen; then, we calculated the mean (*μ*) and standard deviation, *σ*, at each *U* using a probability fit to a Gaussian distribution. Previously, we applied Anderson-Darling tests to verify the null hypothesis to different probability distributions; the Gaussian distribution was the most likely distribution for each dataset.

**Figure 4:**
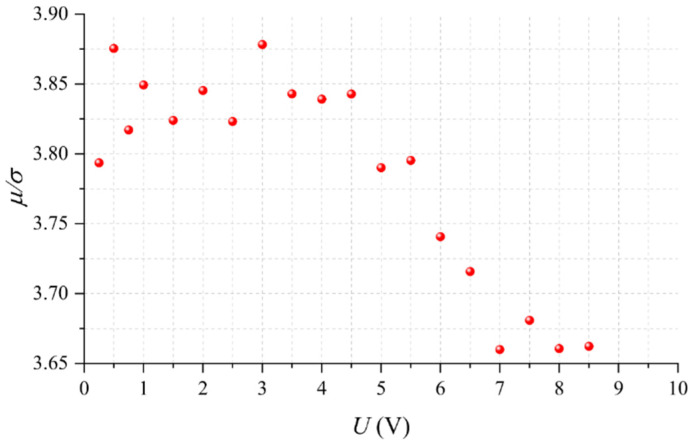
Inverse of the coefficient of variation (*μ/σ*) estimated at different input voltages (*U*) for the 48 sensors under study. Probability fitting to a Gaussian distribution was performed at each *U* to estimate *μ* and *σ*.

As a part of the *improve* stage, the ratio of *μ/σ* as a function of *U* is shown in Figure 4; this chart lets us assess which input voltage naturally yields the highest part-to-part repeatability. The quotient *μ/σ* is known in literature as the inverse of the coefficient of variation. Note that at *U* = 3 V, the dispersion in sensitivity is the lowest. Hence, we can state that when operating Interlink sensors at 3 V, part-to-part repeatability is naturally maximized. We focus on this start point to further reduce *σ*. As previously mentioned, this study comprised the application of compensation techniques at the last stage of final application deployment, therefore, we could only trim *U* to target a desired sensitivity for a given sensor; see claim (iii) from the *Methods* section. In practice, it was possible to change *U* by means of a DAC; see Figure 2.

### 4.1. Sensor Classification on the Basis of the Output Voltage at Null Force

**Figure 5:**
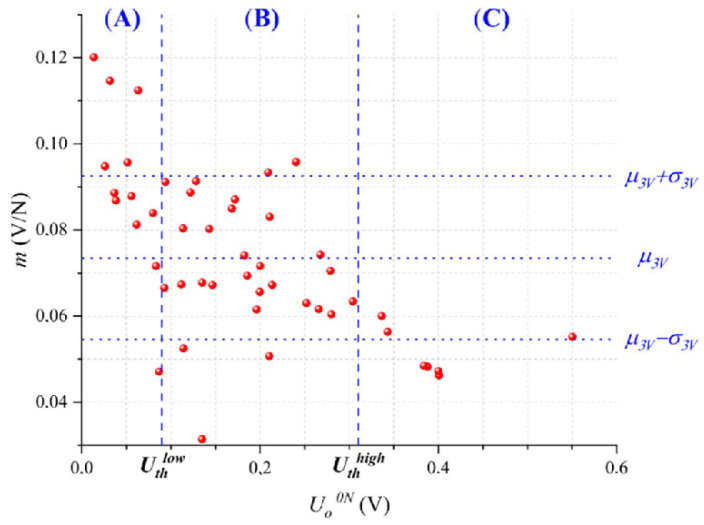
Sensitivity (*m*) as a function of the output voltage at null force (*U_o_^0N^*) for each specimen. The sensitivity data were fit to a Gaussian probability distribution resulting in the mean, *μ_3V_ =* 0.0735 V/N, and standard deviation, *σ_3V_* = 0.019 V/N. Low threshold voltage (*U_th_^low^*) and high threshold voltage (*U_th_^high^*) were defined for classification purposes. Data were taken at *U* = 3 V for 48 sensors.

From the experimental data taken at *U* = 3 V, we plotted in Figure 5 each sensor sensitivity, *m*, with its corresponding output voltage at null force, *U_o_^0N^*. Later, the experimental data were fitted to a Gaussian probability distribution resulting in a mean value of *μ_3V_ =* 0.0735 V/N with standard deviation *σ_3V_* = 0.019 V/N.

As predicted by claim (iv), we observed that sensors exhibiting a large *U_o_^0N^* tend to show a low *m*, and that similarly, sensors with a small *U_o_^0N^* tended to show a large *m*. In other words, *U_o_^0N^* is approximately related to sensor sensitivity. To the best of the authors’ knowledge, this observation has not been published elsewhere in specialized literature, but it represents a powerful tool, as described in the next paragraph. For compensation purposes in the *improve* stage, we have segmented the dataset from Figure 5 into three regions according to *U_o_^0N^*: (A) sensors where quantum tunneling dominates that meet the criteria *U_o_^0N^* < *U_th_^low^*, (B) sensors where quantum tunneling and contact resistance have similar weight, and (C), sensors where contact resistance dominates that meet the criteria *U_o_^0N^* > *U_th_^high^*. The procedure for setting the threshold voltages (*U_th_^low^*, *U_th_^high^*) is described in the next section.

The Importance of *U_o_^0N^* for assessing sensor’s sensitivity is twofold. First, *U_o_^0N^* can be measured at negligible cost after product manufacturing, i.e., a simple driving circuit is required with no mechanical setup, see Figure 2. Second, we can discard non-compliant sensors based on *U_o_^0N^* measurements; this ultimately enhances part-to-part repeatability as described next: by keeping the sensors that meet the criteria *U_th_^high^* < *U_o_^0N^* < *U_th_^low^*, we retain the sensors from Region (B) and discard the sensors from Regions (A) and (C). In practice, this implies that 19 out of the 48 sensors are discarded and 29 are kept; it must be emphasized that the sensors from Region (B) represent our desired target, for this reason, we discard the noncompliant sensors belonging to Regions (A) and (C).

Subsequently, we perform a new fit to a Gaussian distribution from the 29 selected sensors that results in the mean *μ_B_* = 0.0718 V/N with standard deviation *σ_B_* = 0.0142 V/N. Note that *μ_B_* slightly changed from the previously reported value of *μ_3V_* = 0.0735 V/N, but that *σ_B_* decreased 25% from its original value of *σ_3V_* = 0.019V/N. From a Six Sigma standpoint, this represents a 1.5*σ* improvement. Finally, the quotient *μ_B_*/*σ_B_* can be recalculated with the 29 selected sensors as 5.05, which is greater than the value of 3.87 reported in Figure 4. This simple procedure is useful for enhancing part-to-part repeatability, but unfortunately, it occurs at the cost of discarding sensors, which unavoidably represents material waste. Therefore, in the next section, we introduce an alternative compensation technique for the sensors in Regions (A) and (C).

Before proceeding with the compensation technique, we must recall the random distribution of nanoparticles along the polymer matrix. This fact unavoidably causes some specimens from Region (B) to show either a sensitivity larger than *μ_3V_ + σ_3V_* or lower than *μ_3V_ − σ_3V_*; the physical reasons for this behavior are manifold, but we can point out some of them: a given specimen may have a particle count below average; thus, sensitivity is lowered. Another possible reason is the random spacing of clusters that creates isolated conglomerates in the polymer matrix; these separated clusters are just too far from each other to enable quantum tunneling. See Figure 1 for a schematic representation of this.

### 4.2. Compensation Technique to Enhance Part-to-Part Repeatability 

Following the *improve* stage; new driving voltages must be found for the sensors belonging to Regions (A) and (C), see Figure 5; this procedure is supported by claim (iii). By doing this, we can closely match each specimen’s sensitivity with *μ_3V_*, i.e., the target sensitivity.

For the sensors belonging to Region (A), we must find a new *U* that lowers the sensitivity of each specimen. In this case, the new *U* must be lower than 3 V. For the sensors belonging to Region (C), the opposite occurs; the new input voltage must be higher than 3 V to increase their sensitivity. As stated in claim (ii), a change in *U* also modifies *U_o_^0N^*; this is not a problem itself because *U_o_^0N^* can be measured anytime in the final application. 

**Figure 6:**
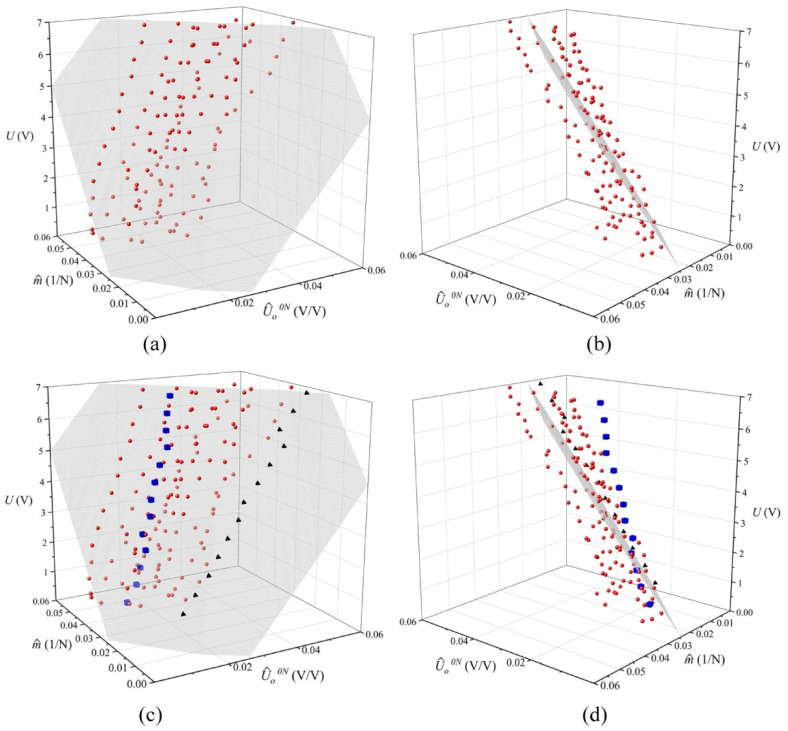
3D plot of the normalized sensitivity ($\hat{m}$), the normalized output voltage at null force (${\hat{U}}_{o}{}^{0N}$ ), and the input voltage (*U*). Data corresponding to the 12 sensors belonging to Region (A). (**a**,**b**) Isometric views of the 3D fit using Equation (8) as the model with parameters shown in Table 2. (**c**,**d**) Isometric views of the 3D fit with special markers. The blue cubes and black pyramid markers correspond to individual sensors that shift in different directions along the plane when *U* is changed.

In order to find a relationship among *m*, *U_o_^0N^*, and *U*, we plotted them together in Figure 6 for the sensors belonging to Region (A). Then, a least-squares fit was applied to find a numerical expression that relates these three variables. The best, yet simplest, function was found to be:

(8) $$U=a\cdot {\hat{U}}_{o}{}^{0N}+b\cdot \hat{m}+c$$

where *a*, *b*, *c* are coefficients resulting from the fit. Normalization was performed for the variables *U_o_^0N^* and *m*, where ${\hat{U}}_{o}{}^{0N}=U_{o}{}^{0N}/U$ and $\hat{m}=m/U$. By doing this, we avoided quadratic functions and used a simple 3D plane resulted instead. The same procedure was repeated for the sensors belonging to Region (C). Both fitting results are summarized in Table 2. The fit to (8) allowed us to determine the optimal threshold voltages for regions (A), (B), and (C). The threshold voltages, *U_th_^low^* and *U_th_^high^*, were previously defined in Figure 5; they let us assess whether quantum tunneling or constriction resistance dominates for a given sensor. The procedure for determining *U_th_^low^* and *U_th_^high^* is described next.

The higher *U_th_^low^* is set, the more sensors are considered as a part of Region (A). However, by doing this, the coefficient of determination (*R*^2^) resulting from the fit is reduced; this occurs because we are embracing sensors that fall out the criteria for this region, i.e., in Region (A) quantum tunneling dominates. The same procedure was repeated to obtain *U_th_^high^*, but in this case, *U_th_^high^* has to be reduced in order to embrace more sensors in Region (C). In practice, *U_th_^low^* and *U_th_^high^* were found by an iterative process that aimed to obtain the largest *U_th_^low^* that minimized *R*^2^ in Region (A), and the smallest *U_th_^high^* that minimized *R*^2^ in Region (C). Finally, *U_th_^low^* and *U_th_^high^* resulted in 90 mV and 310 mV, respectively.

Compensating voltages for the sensors in Region (A) and (C) can also be found from (8). Nonetheless, we cannot directly replace *U_o_^0N^* in (8) and set a targeted sensitivity, *m*, to obtain the new *U*. As previously mentioned, a change in *U* modifies both: *U_o_^0N^* and *m*; see claims (ii), (iii). The 3D surface resulting from (8) can be understood as the plane where *U_o_^0N^* and *m* are shifted when *U* is changed. Nonetheless, every sensor exhibits a different rate of change in *U_o_^0N^* as a function of *U*; this is exemplified with the pyramid and cubic markers in Figure 6. Each marker corresponds to individual sensor data that can be well fitted in the 3D plane, but they move in different angles along the surface when *U* is changed. Therefore, we are limited to obtain an average rate of change for *U_o_^0N^* as a function of *U*; this average comprises the 12 sensors belonging to Region (A). In the Conclusion section, the scenario is discussed when we consider an individual rate of change for each sensor.

Finally, given the mean sensitivity at 3 V for the sensors in Region (A), *μ_A_*, the model from (8), the target sensitivity, *μ_3V_*, and the average rate of change of *U_o_^0N^* as a function of *U,* we computed the new *U* resulting in *U_A_* = 2.5 V. Similarly, the same procedure was performed for the sensors in Region (C), but using *μ_C_* and the average rate of change of *U_o_^0N^* for these sensors; this resulted in the new voltage of *U_C_* = 3.15 V.

**Figure 7:**
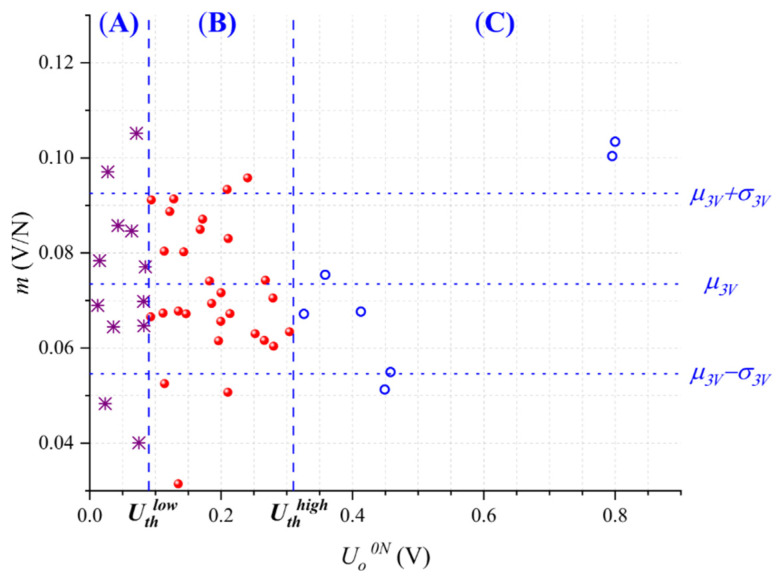
Sensitivity (*m*) as a function of the output voltage at null force (*U_o_^0N^*) for each specimen after compensation. Compensation was performed for the sensors belonging to Regions (A) and (C) using different input voltages (*U_A_*, *U_C_*). For comparison purposes, the y-axis scale was held unchanged from Figure 5. Different markers were used for the sensors of Regions (A) and (C) after compensation.

Later, experimental sensor characterization was performed at the new voltages *U_A_* and *U_C_*; this was done for the sensors belonging to Regions (A) and (C), respectively. The experimental results are plotted in Figure 7. Nonetheless, the sensor data from Region (B) is the same plotted in Figure 5 since *U* remained unchanged for these sensors. For the whole dataset for Figure 7, a new fit to a Gaussian distribution was performed, resulting in *μ_comp_* = 0.0727 V/N and *σ_comp_*= 0.016 V/N. This represents a 15% reduction in the standard deviation when compared with *σ_3V_*. Although this reduction in the standard deviation is lower than previously reported for the discarding method; it is demonstrated that changing *U* is an effective way to fine-tune the sensitivity of FSRs.

### 4.3. Assessing the Compensation Technique from a Six Sigma Perspective

**Figure 8:**
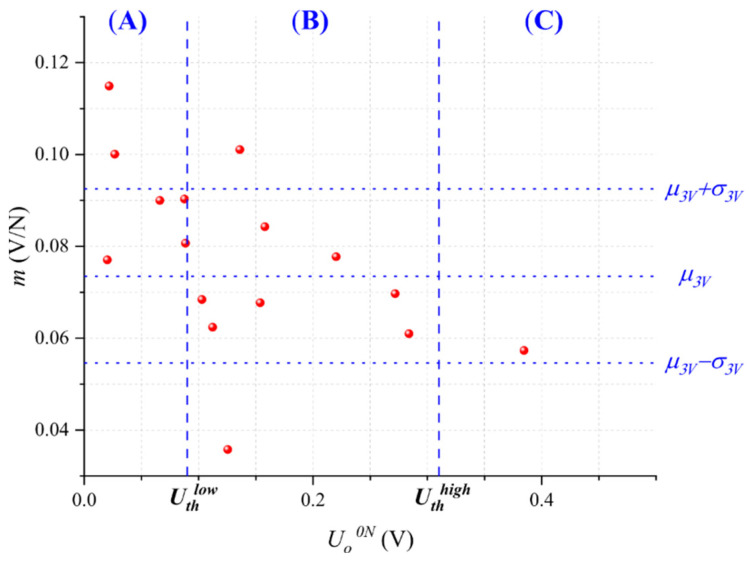
Sensitivity (*m*) as a function of the output voltage at null force (*U_o_^0N^*) for the sixteen specimens of the *control* stage. Data were taken at *U* = 3 V.

As a part of the *control* stage of the DMAIC cycle, we tested an additional group of 16 Interlink FSR 402 sensors. These sensors were initially characterized at *U* = 3 V to obtain *U_o_^0N^* and *m*. Figure 8 shows the summary of this characterization with an overlay of *U_th_^low^*, *U_th_^high^*, *μ_3V_*, and *σ_3V_* for classification purposes. An Anderson-Darling test to different probability distributions demonstrated that these data could be well fitted to a Gaussian distribution with *μ_control_* = 0.0774 V/N and *σ_control_* = 0.0189 V/N. Note that the start point before compensation was quite similar to *μ_3V_* and *σ_3V_* from the *improve* stage.

Later, we classified the sensors belonging to Regions (A), (B), and (C) on the basis of *U_th_^low^* and *U_th_^high^*. Part-to-part repeatability was enhanced following two different approaches: first, by discarding the sensors from Regions (A) and (C) and keeping only those from Region (B), and second, by applying the compensation technique based on changing *U*.

From the first approach, 7 out the 16 sensors were discarded and 9 were kept. Then, we recalculated the mean and standard deviation, obtaining 0.0698 V/N and 0.0168 V/N, respectively; this represents an 11% reduction in the standard deviation and a Six Sigma improvement of 0.66*σ*. However, note that the mean sensitivity was alterated from its original value.

**Figure 9:**
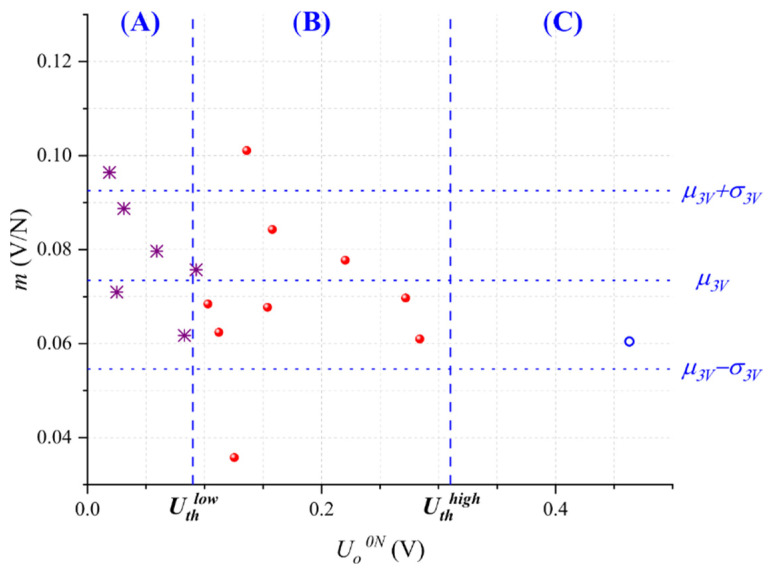
Sensitivity (*m*) as a function of the output voltage at null force (*U_o_^0N^*) for each specimen after compensation. Experimental data resulting from the *control* stage. Different markers were used for the sensors of Regions (A) and (C) after compensation.

From the second approach, new characterization was performed at the input voltages *U_A_* and *U_C_* for the sensors belonging to Regions (A) and (C), respectively. No compensation was performed for the sensors belonging to Region (B). The experimental results are shown in Figure 9. Later, we recalculated the mean and standard deviation, obtaining 0.0726 V/N and 0.0154 V/N, respectively; this represents an 18.5% reduction in the standard deviation amid a negligible variation for the mean sensitivity. This represents a 1.1*σ* improvement using the Six Sigma approach.

### 4.4. Practical Considerations for the Implementation of the Proposed Methods

First of all, it must be emphasized that this research did not consider lot control for the specimens characterized. Therefore, if the manufacturing conditions remain unchanged over time (e.g., particle dimension, type of polymer, preparation conditions, and so on), the compensation techniques should work for any specimen of Interlink FSR 402. In regard to the feedback resistor, a change in *R_f_* produces a linear variation in the magnitude of *m*, *U_o_^0N^*, *U_th_^low^* and *U_th_^high^*; this occurs because the amplifier is an inherently linear device. Therefore, if *R_f_* is doubled or halved, the aforementioned magnitudes also change proportionally.

Nonetheless, a change in the driving voltage would impact *m*, *U_o_^0N^*, *U_th_^low^*, and *U_th_^high^* in a nonlinear fashion; this is expected because quantum tunneling is a nonlinear phenomenon. Besides, we must recall that during the *improve* stage, we found *U* = 3 V as the optimal voltage that naturally maximizes the quotient *μ/σ*; see Figure 4. Therefore, we set *U* = 3 V as the starting point to subsequently trim the input voltage in the *improve* and *control* stages. However, if we set a different starting point for *U*, we would deviate from the results reported here.

Finally, given the relatively low number of sensors considered in this research (64 specimens), only three regions were defined. By considering more regions, a more suitable compensating voltage could be applied for each specimen. The experimental data employed in this study can be found in [56].

## 5. Conclusions

Changing the input voltage is an effective way to trim the sensitivity of force-aensing resistors (FSRs). The output voltage at null force (*U_o_^0N^*) can give us a hint about the individual sensor’s sensitivity without requiring individual characterization. To the best of authors’ knowledge, this observation has not been reported elsewhere. With the information provided from *U_o_^0N^*, we could set new input voltages that let us reduce the dispersion in sensors’ sensitivity and/or discard noncompliant sensors. Both methods were explored in this study, resulting in a reduction of 18.5% and 11% in the standard deviation of sensor sensitivity for each procedure, respectively. These results were obtained in the *control* stage of the DMAIC cycle for a bunch of 16 Interlink FSR 402 sensors. These percentages represent 1.1*σ* and 0.66*σ* improvements according to the Six Sigma methodology.

A promising technique to be explored in the authors’ future work is based on individually defining new input voltages for each sensor; by doing this, we can more accurately match the sensitivity of each specimen with a target sensitivity, thus achieving a larger Six Sigma improvement. However, this procedure requires that we previously track *U_o_^0N^* for multiple input voltages and later incorporate such information in the final compensation formula.

## Figures and Tables

**Table 1:** Summary of the DMAIC cycle with a brief description of each step.

Step of the Cycle	Description
Define	Sensitivity (*m*) of 64 specimens of commercial FSRs, model Interlink FSR402 [27]. A total of 48 sensors were considered for the DMAI stages and 16 for the C stage.
Measure	Sensitivity was measured in force steps of 1 N, starting at 0 N up to 20 N. A total of 19 input voltages (*U*) were considered: 0.25 V, 0.5 V, 0.75 V, and 1 V. Above 1 V, voltage increments of 0.5 V were applied up to 8.5 V.
Analyze	Evaluation of the experimental data in perspective of the underlying physics of FSRs. Four claims were stated to ease the analysis and to derive conclusions.
Improve	The improve step comprised two stages: finding the optimal input voltage that minimizes dispersion in sensitivity, proposing and test two different methods to reduce the dispersion in sensitivity.
Control	Validate the two methods developed in the improve stage using 16 sensors.

**Table 2:** Parameters resulting from the fit. Parameters *a* through *c* were obtained from a least-squares fit to (8) with coefficient of correlation *R*^2^. The mean sensitivity (*μ*) for each region (*μ_A_, μ_C_*) was measured at *U* = 3 V.

	*a* (V)	*b* (N·V)	*c* (V)	*R* ^2^	*μ* (V/N)
Region (A)	132.3	139.1	−3.36	0.67	*μ_A_* = 0.091
Region (C)	101	485.2	−18.2	0.94	*μ_C_* = 0.052

## Data Availability

Experimental data can be found here [56].

## Appendix A. Foundations of the Six Sigma Methodology

Six Sigma is a continuous improvement methodology that has been widely applied in the manufacturing and services sectors. Its implementation focuses on the application of the DMAIC cycle (Define, Measure, Analyze, Improve, and Control) [41]. This methodology is based on the reduction of the process variability regarding a Critical to Quality (CTQ) or critical variable for the process or the client. Process variability is assessed on the basis of the standard deviation (*σ*) for any process parameter, e.g., when building antennas on a printed circuit board (PCB), the width and length of the paths have a strong influence on the antennas’ bandwidth and frequency response [57]; therefore, we can deploy the DMAIC cycle to reduce the dispersion of the width and length of paths from their nominal value.

Six Sigma requires the collection of data and the application of analytical and statistical tools to identify the causes of the variation and thus achieve a 6σ level that translates into 3.4 errors per million opportunities [41]. A 6σ level implies that standard deviation is reduced by a factor of 6 from the original assessment. The opportunity is understood as the probability of noncompliance or the probability of not meeting the required specifications. The DMAIC cycle implemented by the Six Sigma methodology is used to identify and solve problems related to the process; analyzing the current state of a problem of interest, find its root cause, propose improvement solutions, and keep them durable over time.
